# Stratified Randomized Controlled Trial of ∅3.3 mm Versus ∅4.1 mm Hydrophilic Titanium‐Zirconium Implants Supporting Posterior Mandibular Fixed Prostheses in Hyperglycaemic Subjects

**DOI:** 10.1111/clr.70175

**Published:** 2026-09-24

**Authors:** Supriya Ebenezer, Sreelakshmi Viswanath, Chitradevi Ramanathan, Alampalli V. Ramesh, Vinay V. Kumar

**Affiliations:** ^1^ Oral Rehabilitation Center Bangalore India; ^2^ Sankara Nethralaya Medical Research Foundation Chennai India; ^3^ Dental Health Clinic Bangalore India; ^4^ Department of Odontology Maxillofacial Surgery, Division of Surgical Sciences Uppsala University Uppsala Sweden; ^5^ Coorg Institute of Dental Sciences Karnataka India; ^6^ Department of Maxillofacial Surgery and Dentistry Vydehi Superspeciality Hospital Bangalore India

**Keywords:** alveolar bone loss, dental implants, glycated haemoglobin, osseointegration, titanium, Type 2 diabetes mellitus, zirconium

## Abstract

**Objective:**

To compare ⌀3.3 mm and ⌀4.1 mm hydrophilic titanium‐zirconium implants supporting fixed dental prostheses (FDPs) in mandibular distal‐extension situations among participants with varying degrees of hyperglycaemia.

**Materials and Methods:**

Participants were stratified according to HbA1c (5.8%–< 7.0% vs. ≥ 7.0%) and subsequently randomized within each stratum to receive either ⌀3.3 mm or ⌀4.1 mm titanium‐zirconium implants. Each participant received a two‐implant‐supported multi‐unit FDP. The primary outcome was marginal bone level (MBL) change at 12 months after prosthesis delivery. Secondary outcomes included implant stability quotient (ISQ), peri‐implant indices, Healing Index and patient‐reported outcomes.

**Results:**

Of 88 enrolled participants, 83 (166 implants) were evaluated. Implant survival was 100%, with no peri‐implantitis. At 12 months, MBL did not differ significantly between the ⌀3.3 mm [0.27 mm (IQR, 0.07–0.68)] and ⌀4.1 mm groups [0.27 mm (IQR, −0.04 to 0.54); *p* = 0.078]. Participants with HbA1c ≥ 7% had approximately sevenfold higher odds of MBL ≥ 0.5 mm than those with HbA1c < 7%. Secondary ISQ was significantly higher with ⌀4.1 mm implants (79.6 vs. 76.1; *p* < 0.001), whereas early healing was better with ⌀3.3 mm implants (*p* = 0.005). Chewing ability improved significantly from baseline to 12 months irrespective of implant diameter or HbA1c category (*p* < 0.0001).

**Conclusion:**

At 1 year, ⌀3.3 mm and ⌀4.1 mm hydrophilic Ti‐Zr implants demonstrated no statistically significant difference in MBL although ⌀4.1 mm implants demonstrated higher stability. Patient‐reported chewing ability improved substantially following rehabilitation for both implant diameters. Higher HbA1c was associated with greater odds of clinically relevant MBL.

**Trial Registration:**

The trial was registered prospectively with the Clinical Trials Registry–India (CTRI/2017/08/009315)

## 1 Introduction

Dental implant diameter is a key determinant of primary stability, biomechanical load distribution, peri‐implant bone preservation and long‐term clinical success, particularly in posterior mandibular regions where occlusal forces are considerably high. Regular‐diameter implants (⌀4–⌀5 mm) have traditionally been regarded as the preferred choice for posterior rehabilitation because of their greater bone‐to‐implant contact area, improved stress distribution and reduced susceptibility to mechanical complications such as implant fracture or prosthetic screw loosening. However, narrow diameter implants offer a valuable treatment alternative by reducing the need for augmentation, thereby reducing treatment morbidity, cost and healing time (Ioannidis et al. 2015; Klein et al. 2014; Schiegnitz and Al‐Nawas 2018).

Titanium‐zirconium (Ti‐Zr) alloy implants with hydrophilic surface modifications have expanded the clinical applicability of reduced‐diameter implants by combining high mechanical strength with favourable biocompatibility and enhanced early osseointegration (Buser et al. 2004; Medvedev et al. 2016; Saulacic et al. 2012; Sawai et al. 2024), which may be particularly relevant in participants with diabetes mellitus where wound healing and bone metabolism can be impaired.

Type 2 Diabetes Mellitus (T2DM) is among the top four priority noncommunicable diseases in the world (World Health Organization (WHO) 2016). In 2022, an estimated 828 million adults had T2DM, an increase of 630 million from 1990 (NCD Risk Factor Collaboration 2024), and the prevalence is expected to double by the year 2030 (Kaveeshwar and Cornwall 2014). Management of T2DM is primarily monitored through glycated haemoglobin (HbA1c) levels, with the aim to achieve HbA1c below 7% (American Diabetes Association 2013). The American Diabetes Association (2020) suggests that lowering HbA1c to below 7% reduces microvascular complications and is considered a therapeutic goal for non‐pregnant adults. Chronic hyperglycaemia is known to impair wound healing, alter inflammatory responses, reduce osteoblastic activity and compromise bone remodelling, thereby increasing the risk of delayed osseointegration, increased peri‐implant complications and implant failure. Diabetics are prone to periodontitis and tooth loss (Heitz‐Mayfield 2005; Weijdijk et al. 2022; Yoo et al. 2019), and form an increasing patient pool for restoring edentulousness.

Although dental implants have demonstrated favourable outcomes in subjects with controlled diabetes (Khandelwal et al. 2013; Oates et al. 2009; Oates Jr. et al. 2014), HbA1c ≥ 7 has been considered a relative contraindication, or a potential risk factor for implant therapy because of concerns regarding osseointegration and delayed healing (Glowacki 1998) and higher infection risks (Morris et al. 2000; Moy et al. 2005). Consequently, elective surgeries, such as implant therapy, in diabetic subjects are often deferred until glycaemic control is achieved (American Diabetes Association 2020). The use of less invasive, narrow diameter implants could serve as a promising risk mitigation strategy.

Clinical studies have demonstrated the success of ⌀3.3 mm hydrophilic Ti‐Zr implants in applications ranging from implant‐supported overdentures to single molar replacements (Al‐Nawas et al. 2015; Altinci et al. 2016; Badran et al. 2017; Brignardello‐Petersen 2017; Ioannidis et al. 2015; Jung et al. 2018; Muller et al. 2015; Quirynen et al. 2015; Zembic et al. 2016). However, there is limited research on their use in mandibular posterior edentulous spans supporting fixed bridges in distal extension situations.

This study aims to evaluate the clinical success of ⌀3.3 mm hydrophilic Ti‐Zr implants compared to ⌀4.1 mm hydrophilic Ti‐Zr implants in supporting fixed dental prostheses in mandibular distal extension situations of subjects with varying levels of hyperglycaemia. The study evaluated implant survival, stability, peri‐implant marginal bone changes, peri‐implant soft tissue parameters and patient‐reported outcomes according to implant diameter and HbA1c category. The hypothesis was that ⌀3.3 mm implants would demonstrate clinical and radiologic outcomes similar to those of ⌀4.1 mm implants across HbA1c categories.

## 2 Materials and Methods

### 2.1 Study Design, Settings and Ethical Considerations

This prospective randomized controlled clinical study was conducted with equal allocation. The study protocol is presented in Figure 1, and the PICOT framework is presented in Table S1. The study was conducted at two tertiary care, university‐affiliated dental clinics: Dental Health Centre, Chamarajpet, Bangalore, and Oral Rehabilitation Center, Bangalore, India. Despite recruitment at two centres, the entire study team, including the surgeon, restorative dentists, dental laboratory, and outcome evaluators, remained the same. The study was approved by the respective ethics committees and institutional review boards (NHH/AEC‐CL‐2017‐184; IEC‐AIMS‐2018‐DENT‐089; VDC/IEC/2019/26). Participants received clear instructions regarding the nature of the study and the importance of glycaemic control in preventing complications after implant placement. All participants provided written informed consent, and the study was conducted in accordance with the Declaration of Helsinki (2013). The trial was prospectively registered with the Clinical Trials Registry–India (CTRI/2017/08/009315). Data were collected in pseudonymized form, and participants' names did not appear on any study‐related case report form or other trial document submitted for data management. All research activities and reporting were conducted in accordance with CONSORT guidelines (Schulz et al. 2010).

**FIGURE 1:**
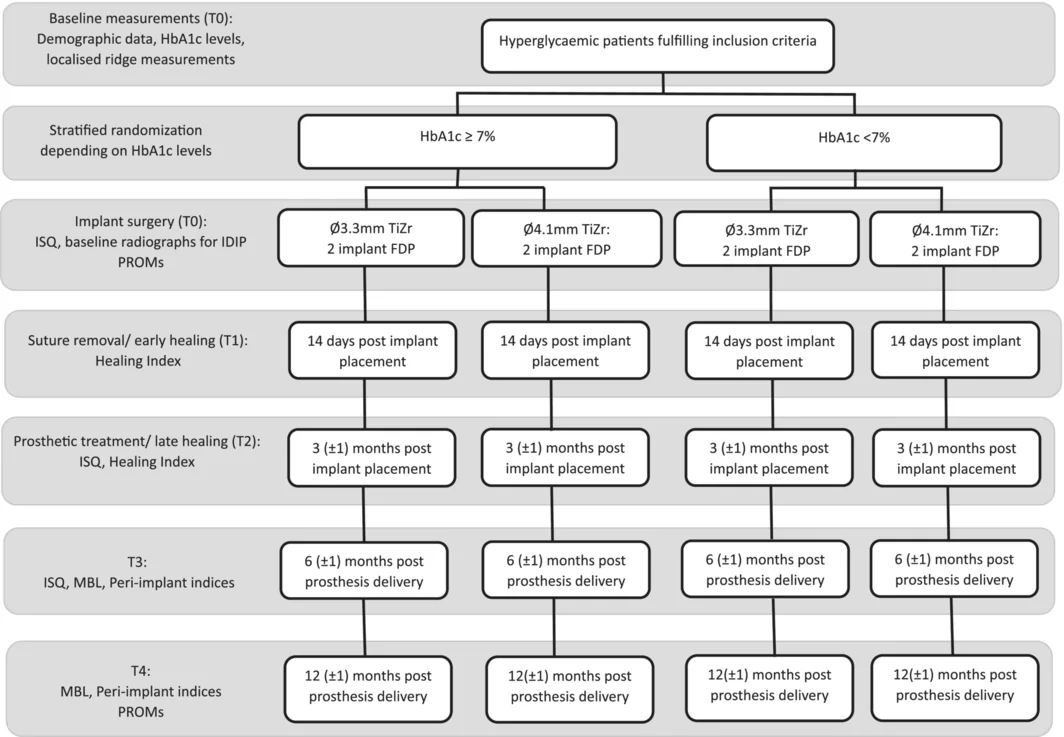
Describes the study protocol, stratification, randomization and the outcome measures at corresponding timelines of the study. FDP, fixed dental prosthesis; HbA1c, glycated haemoglobin; IDIP, initial depth of implant placement; ISQ, implant stability quotient; MBL, marginal bone level changes; PROMs, patient reported outcome measures.

### 2.2 Study Population

The inclusion criteria for the study were as follows:

- Participants with known history of pre‐diabetes or diabetes, with documented HbA1c levels (minimal 5.8%, no maximal limit).
- Posterior mandibular free end situation (third molars not counted) with loss of at least two posterior teeth, requiring a fixed dental prosthesis.
- Presence of opposing natural dentition or tooth‐supported crown/bridge.
- Age of more than 18 years at the beginning of treatment.
- Sufficient bone height from the inferior alveolar canal to the crestal ridge to place an implant of at least 8 mm length (bone height of 9.5 mm).
- Sufficient bone width to place at least ⌀3.3 mm implant in a prosthetically driven implant position (minimum width of 5.3 mm).
- Adequate amount of keratinized oral mucosa (minimum 2 mm thickness) in the edentulous ridge.
- Participants who are willing to participate and have signed the informed consent form.

The exclusion criteria for the study were as follows:

- Participants with a concomitant history of other systemic medical disorders that do not permit implant treatment: such as prolonged use of steroids, severe haemophilia, bisphosphonate medication, leukocyte dysfunction or deficiencies, therapeutic radiation to the head and neck region, ongoing anticancer chemotherapy, renal failure, uncontrolled endocrine disorders other than diabetes mellitus, differently abled physical or mental conditions that interfere with the ability to perform oral hygiene or use of any investigational drugs or device within 30 days prior to implant surgery.
- Alcoholism, drug abuse, smoking > 10 cigarettes per day, chewing arecanut or chewing tobacco in excess of 3 packets a day.
- Localized exclusion criteria included mucosal diseases such as erosive lichen planus, presence of osseous or soft tissue pathologies, active periodontal disease and acute infection at/around the site of implant placement.
- Participants who are non‐compliant with oral hygiene maintenance.
- Presence of edentulousness, complete denture, removable denture or implant‐supported prosthesis to replace the opposing maxillary teeth.

### 2.3 Study Grouping and Randomization

Following enrolment, baseline HbA1c levels were measured by laboratory analysis. Based on these measurements and the American Diabetes Association criteria (American Diabetes Association 2013), participants were stratified into two groups: those with HbA1c ≥ 7.0% (designated as the exposed group) and those with 5.8% < HbA1c < 7.0% (designated as the unexposed group).

Within each HbA1c stratum, participants were randomly assigned in a 1:1 ratio to receive either ⌀3.3 mm implants (test) or ⌀4.1 mm implants (control) using computer‐generated randomization with a block size of four (www.researchrandomiser.org). The randomization sequence, participant enrolment, and group assignment were performed by VVK. The allocation code was sealed in an envelope and opened only after participant inclusion. Participants were assigned to the respective groups according to the concealed allocation sequence. Participants, operators and assessors were not blinded to the treatment received (www.researchrandomiser.org).

This resulted in four subgroups (Figure 1): ⌀3.3 mm implants with HbA1c ≥ 7% (test‐exposed), ⌀4.1 mm implants with HbA1c ≥ 7% (control‐exposed), ⌀3.3 mm implants with HbA1c < 7% (test‐unexposed) and ⌀4.1 mm implants with HbA1c < 7% (control‐unexposed).

The minimum ridge width specified in the inclusion criteria was 5.3 mm; therefore, all participants allocated to ⌀3.3 mm implants had sufficient width without augmentation. For ⌀4.1 mm implants, the minimum required width was 6.1 mm. Accordingly, participants allocated to ⌀4.1 mm implants with a ridge width of 5.3–6.1 mm were offered simultaneous guided bone regeneration (GBR) at implant placement. Participants who declined augmentation were excluded from the per‐protocol analysis, and the next eligible participant was assigned according to the predefined randomization sequence.

The primary analysis followed a ‘per‐protocol approach’ (Smith et al. 2021), as prespecified for the treatment comparison.

### 2.4 Intervention

#### 2.4.1 Implant Insertion

After being allocated to the respective randomized groups, implants were inserted as per the manufacturer's protocol, by a single clinician (V.V.K.). For participants with ⌀3.3 mm implants, two ⌀3.3 mm (8 or 10 mm length, Straumann Roxolid Bone Level Tapered SLActive implant; Institut Straumann AG, Basel, Switzerland) were placed. For participants with ⌀4.1 mm implants, two ⌀4.1 mm (8 or 10 mm length, Straumann Roxolid Bone Level Tapered SLActive implant; Institut Straumann AG, Basel Switzerland) were placed. All implants were placed freehand in a prosthodontically driven position.

Since all participants were hyperglycemic, precautions were taken to ensure patient safety. All participants received postoperative care instructions and were prescribed antibiotics for 1 week (Amoxycillin with Clavulanic acid), appropriate analgesics (Paracetamol and Ketorolac as required) as well as Chlorhexidine 0.2% mouth rinses for 2 weeks. Emphasis was placed on the importance of maintaining both optimal oral hygiene and glycemic control. They were advised to immediately report to the PI telephonically should they notice any untoward event.

At implant placement, if primary implant stability, measured as the implant stability quotient (ISQ), was ≥ 65 (as described below), a transmucosal healing protocol was followed. For implants with ISQ < 65, a submerged healing protocol was used. Participants were recalled 2 weeks after surgery for suture removal and evaluation of wound healing.

#### 2.4.2 Prosthetic Restoration

The prosthetic phase was initiated at the 3‐month time point following implant placement in participants who underwent transmucosal healing. For participants in whom the submerged healing protocol was followed, healing abutments were placed at the 3‐month time point, and the prosthetic phase was started 2 weeks following the placement of the healing abutment.

All participants received splinted, screw‐retained fixed prosthetic restorations. Depending on the number of units replaced, either a two‐unit or a three‐unit bridge supported by two implants was provided.

Open tray impressions were recorded using appropriate impression posts and elastomeric impression material, the bite and shade were recorded and sent to the laboratory for the fabrication of porcelain fused to metal fixed partial denture on titanium abutments (Straumann Variobase, Institut Straumann AG, Basel, Switzerland). The clinical prosthetic steps included a jig trial to verify the fit, followed by metal try‐in, followed by final try‐in. Occlusal and proximal contacts were checked prior to finalizing the restoration. Clinical prosthetic procedures were done by either V.V.K., S.E. or A.V.R. All laboratory procedures were done at a single laboratory (VIP Dental Lab, Bangalore, India). The restorations were screw‐retained onto the implants with 35 N/cm torque. The screw access hole was covered with PTFE tape followed by closure of the screw channel with light‐polymerizing resin‐based restorative material, as described prior (Moraguez and Belser 2010). Occlusal and proximal contacts were inspected to confirm optimal occlusion.

### 2.5 Outcome Assessments

Baseline characteristics evaluated at the time of recruitment included demographic characteristics (age and sex), glycated haemoglobin levels, teeth that are to be replaced according to the FDI World Dental Federation (FDI system), and the width and length of the available bone at the region of implant placement.

#### 2.5.1 Follow‐Up Periods

The evaluation of subjects was carried out at several key intervals throughout the course of the study (Figure 1). The initial assessment, referred to as T0, took place at the time of implant placement. During this stage, OHIP‐14 and chewing ability using numerical rating scale were recorded prior to the procedure, and ISQ and the Initial Depth of Implant Placement (IDIP) were recorded immediately after the implants were inserted.

Two weeks (14 days) following the implant placement, at T1, the Healing Index (HI) was assessed to monitor the early stages of wound healing. The next significant review occurred at 3 months post‐placement (T2), during which both the ISQ and HI were measured again to evaluate secondary stability and healing.

Six months after the prosthesis had been delivered, the T3 evaluation was conducted. This appointment focused on assessing marginal bone level (MBL) changes and examining peri‐implant indices to monitor the health of the surrounding tissues and bone. Finally, at the 12‐month mark following prosthesis delivery (T4), a comprehensive review was performed. This included reassessment of MBL, peri‐implant indices, and a repeat of OHIP‐14 and the numerical rating scale to assess chewing ability, to gain insight into both clinical and patient‐centred outcomes.

Figure 2A–D shows clinical and radiologic pictures representing the 4 subgroups of the study.

**FIGURE 2:**
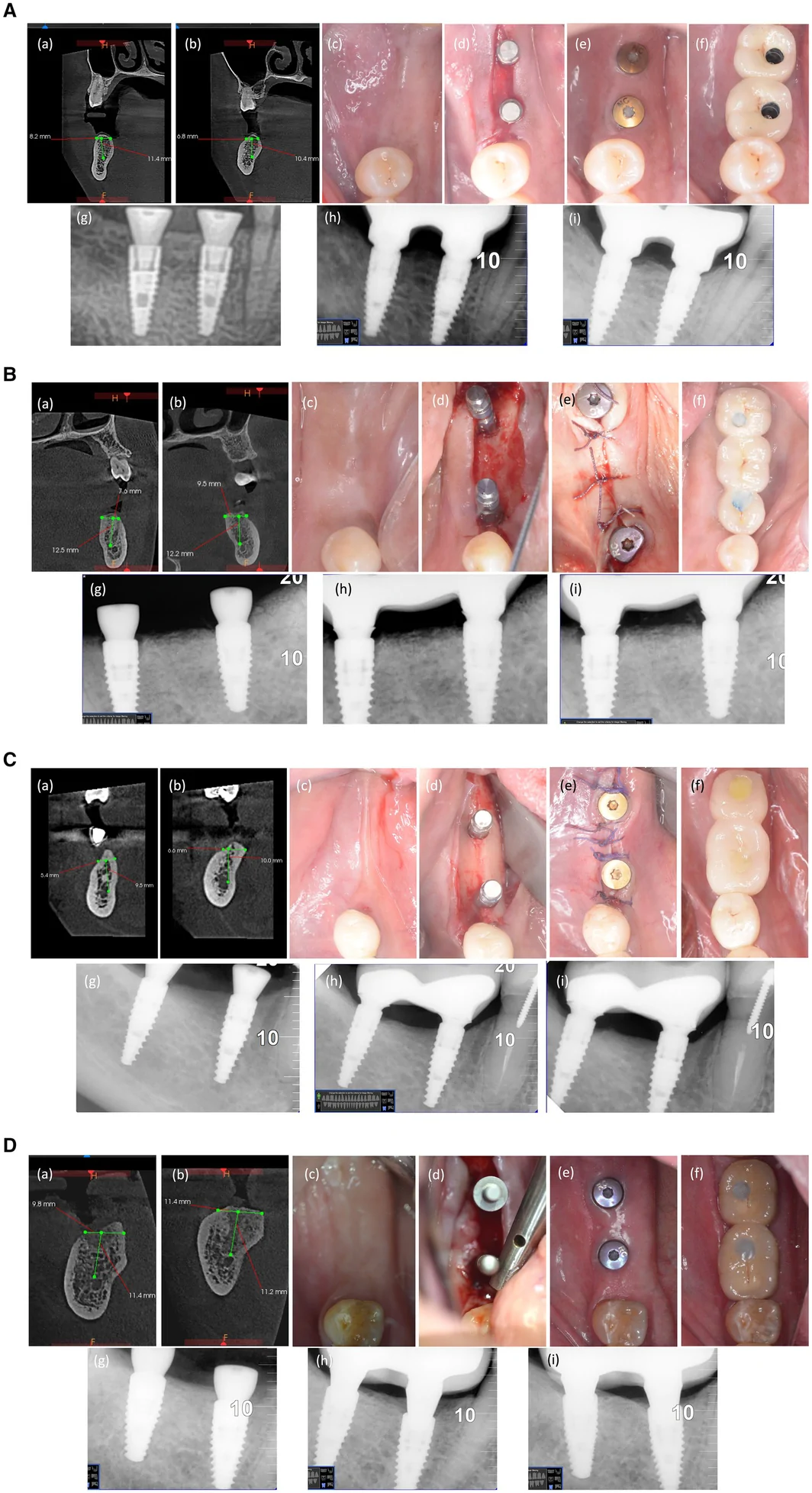
Radiographic and clinical representation of a case from each group. (A) Radiographic and clinical representation of Group ⌀3.3 mm implants with HbA1c ≥ 7%. (a) CBCT width and length measurements for site 1; (b) CBCT width and length measurements for site 2; (c) pre‐operative site; (d) flap reflection with implant placement; (e) healed site with NC healing abutments; (f) final prosthesis; (g) T0 implant radiograph; (h) T3 radiograph; (i) T4 radiograph. (B) Radiographic and clinical representation of Group ⌀4.1 mm implants with HbA1c ≥ 7%. (a) CBCT width and length measurements for site 1; (b) CBCT width and length measurements of site 2; (c) pre‐operative site; (d) flap reflection with implant placement; (e) sutured site with RC healing abutments; (f) final prosthesis in place; (g) T0 implant radiograph; (h) T3 radiograph; (i) T4 radiograph. (C) Radiographic and clinical representation of Group ⌀3.3 mm implants with HbA1c < 7%. (a) CBCT width and length measurements for site 1; (b) CBCT width and length measurements of site 2; (c) pre‐operative site; (d) flap reflection with implant placement; (e) healed sutured site with NC healing abutments in place; (f) Final prosthesis; (g) T0 implant radiograph; (h) T3 radiograph; (i) T4 radiograph. (D) Radiographic and clinical representation of Group ⌀4.1 mm implants with HbA1c < 7%. (a) CBCT width and length measurements for site 1; (b) CBCT width and length measurements of site 2; (c) pre‐operative site; (d) flap reflection with implant placement; (e) healed site with RC healing abutments in place; (f) final prosthesis; (g) T0 implant radiograph; (h) T3 radiograph; (i) T4 radiograph.

Success criteria for the implants followed the Buser criteria (Buser et al. 1990).

#### 2.5.2 Primary Outcome (MBL)

The primary outcome measure was radiographic marginal bone level change (MBL) at the 12‐month follow‐up time point.

Radiographic measurements of marginal bone were done using digital standardized periapical radiographs by using the paralleling technique using positioners, processed and analysed following the methods described previously (Ioannidis et al. 2015). The reference points for the measurements were the implant platform (the horizontal interface between the implant and the abutment) and the first bone‐implant contact (Kumar et al. 2014). Measurements were made both mesially and distally. The Initial Depth of Implant Placement (IDIP) was measured in mm using the immediate post‐implantation radiograph at T0. MBL was calculated as the difference between the marginal bone level at each follow‐up time point and the initial depth of implant placement (IDIP) measured on the immediate post‐placement radiograph. Positive values represented bone loss and negative values represented bone gain. The follow‐up radiographs were recorded at 6 months and 12 months following prosthesis delivery.

Radiographs were analysed with an image analysing software (Image J 1.4q, National Institute of Health, USA). Implant depth measurements were taken for each implant individually by magnifying the radiographic picture whereby the level of crestal bone was assessed and measured at the mesial and distal margins of the implant. All measurements were recorded by two examiners (S.E. and S.V.) who evaluated the radiographs together, discussed the measurements and then arrived at agreeable values.

#### 2.5.3 Secondary Outcomes

##### 2.5.3.1 Implant Stability

Implant stability was determined by Implant Stability Quotient (ISQ) via Resonance Frequency Analysis (RFA) as described previously (Kumar et al. 2017). A MentorTM instrument (Osstell AB, Göteborg, Sweden) was used to make magnetic RFA measurements according to the manufacturer's protocol. A compatible SmartpegTM (No. 53 for ⌀3.3 mm and No. 54 for ⌀4.1 mm) was inserted manually into the internal connection of the implant and tightened. The measurements were performed thrice both vertically as well as laterally, and a mean of these six values was considered. ISQ values were measured at baseline and 3 months post‐placement.

##### 2.5.3.2 Clinical Peri‐Implant Parameters

Clinical evaluation of peri‐implant health using indices to evaluate plaque, gingival inflammation (Mombelli et al. 1987), measurement of probing depth, peri‐implant mucositis and peri‐implantitis (Berglundh et al. 2018).

All peri‐implant indices and probing depth were measured at four sites (mesial, labial, distal and lingual) per implant. Probing Depth (PD) was measured using a periodontal probe (Click‐Probe, KerrHawe S.A., Switzerland) with 20–25 g probing force.

Peri‐implant mucositis and peri‐implantitis were defined as per the criteria defined by Berglundh et al. (2018).

All clinical peri‐implant soft‐tissue measurements were performed at 6 and 12 months after prosthesis delivery by a single experienced periodontist (S.E.).

##### 2.5.3.3 Assessment of Wound Healing

Wound healing was assessed using the Healing Index (HI) of Landry and Howley (1988) and Masse et al. (1993) at 14 days and 3 months after implant placement. Table 1 presents the HI with clinical examples from the study.

**TABLE 1:** Elaboration of Healing Index by Landry and Howley (1988) and Masse et al. (1993) using clinical examples from the study.

Indices	Healing index	Tissue colour	Response to palpation	Granulation tissue	Incision margin	Clinical example
1	Very poor	≥ 50% gingiva is red	Bleeding	Present	Not epithelialized, with loss of epithelium beyond wound margin; Suppuration present	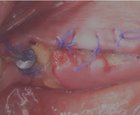
2	Poor	≥ 50% gingiva is red	Bleeding	Present	Not epithelialized, with connective tissue exposed	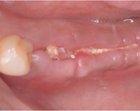
3	Good	≥ 25% and < 50% of gingiva is red	No bleeding	None	No connective tissue exposed	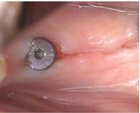
4	Very good	< 25% of gingiva is red	No bleeding	None	No connective tissue exposed	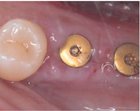
5	Excellent	All tissue is pink	No bleeding	None	No connective tissue exposed	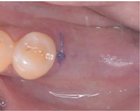

##### 2.5.3.4 Patient Reported Outcome Measures (PROMs)

PROMs were recorded using the English version of OHIP‐14 QoL questionnaire (Slade 1997) at baseline and at 12 months post‐prosthetic loading. OHIP‐14 is a well‐validated questionnaire consisting of 14 questions that measures oral function in 7 dimensions of oral health‐related quality of life that include functional limitation, physical pain, psychological discomfort, physical disability, psychological disability, social disability and handicap. The participants were asked to mark their answers to each question based on a 5‐point Likert Scale from 0 to 4 (0 = never, 1 = hardly ever, 2 = occasionally, 3 = fairly often and 4 = very often). It has been previously used in similar settings (Kumar et al. 2016).

In addition, a numerical rating scale for chewing ability, ranging from 0 to 10 (0 = I feel I cannot chew any food, 10 = I feel I can chew any food I want), was used to report participants' satisfaction in eating various types of food at both time points.

### 2.6 Adverse Events, Technical and Prosthetic Complications

The occurrence of adverse events and technical and prosthetic complications was recorded from the time of surgery until 12 months after prosthesis delivery.

### 2.7 Sample Size Estimation

The original prespecified sample‐size calculation used implant‐level 12‐month MBL, assuming a between‐group difference of 0.5 mm, standard deviation 0.7 mm, *α* = 0.05 and 80% power across four diameter/HbA1c groups. This yielded 32 implants per group; allowing 20% attrition resulted in a target of 40 implants, or 20 participants, per group. Because clustering of two implants within each participant was not explicitly incorporated into the original calculation, a subsequent sensitivity calculation assuming an ICC of 0.10 yielded a similar requirement. This latter calculation was performed after the original calculation and is reported transparently as a sensitivity analysis rather than as the prespecified basis of sample size.

### 2.8 Causal Framework and Covariate Selection

A directed acyclic graph (DAG), developed and shown in Figure 3, guided covariate selection. HbA1c category (< 7% vs. ≥ 7%) and implant diameter (3.3 vs. 4.1 mm) were defined as the main exposures, with age, sex, and baseline ridge width selected as prespecified adjustment variables. The primary outcome was marginal bone loss (MBL); secondary outcomes included ISQ, peri‐implant indices, peri‐implant mucositis, healing index, and MBL ≥ 0.5 mm at 6 and 12 months. Potential HbA1c–diameter effect modification was explored through stratified analyses.

**FIGURE 3:**
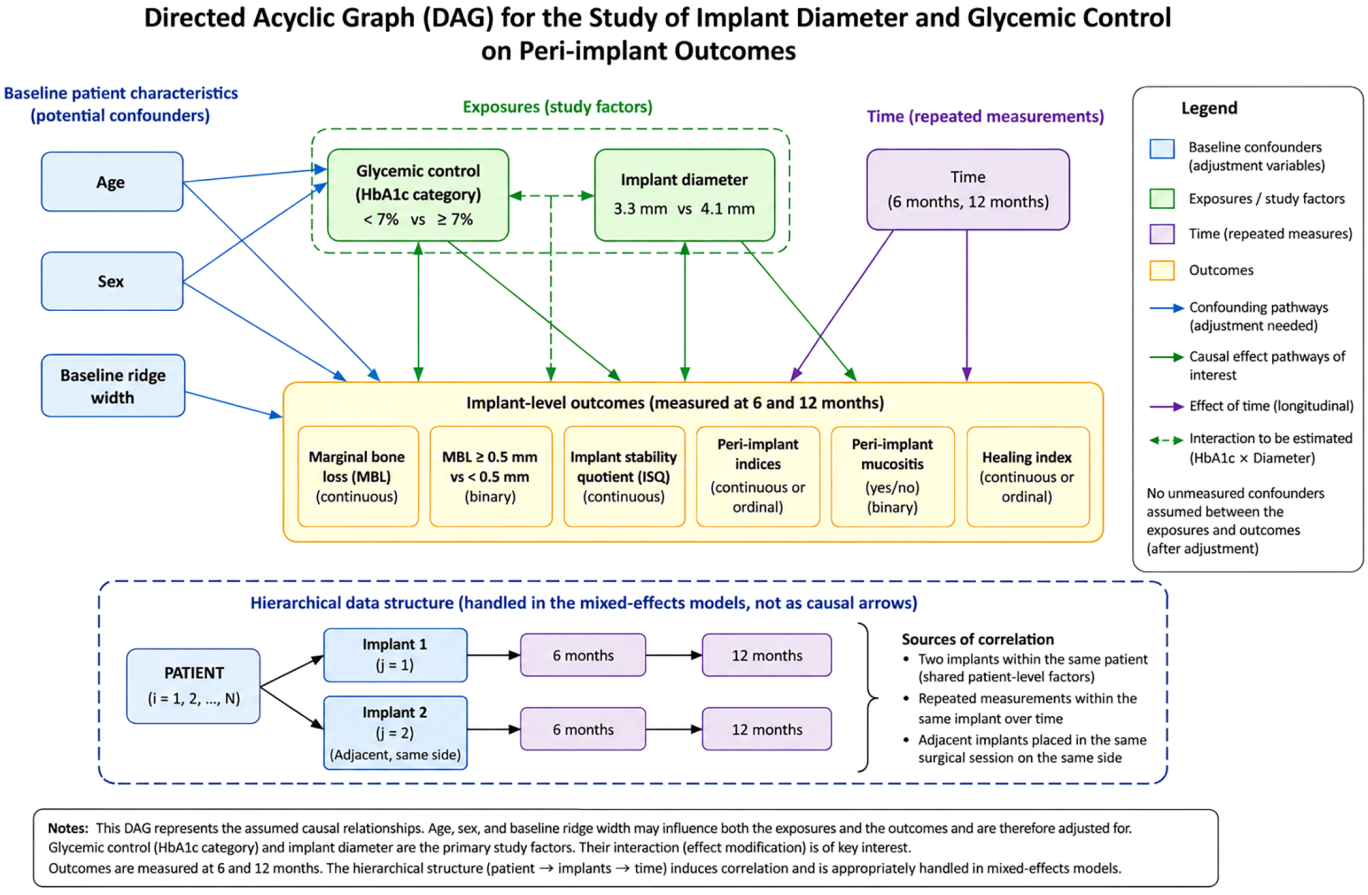
Directed acyclic graph (DAG) illustrating the theoretical framework for the association between glycemic control, implant diameter and peri‐implant outcomes. The DAG identifies HbA1c category (< 7% vs. ≥ 7%) and implant diameter (⌀3.3 vs. ⌀4.1 mm) as the principal study factors and depicts age, sex and baseline ridge width as prespecified baseline variables considered in adjustment. The interaction between HbA1c category and implant diameter represents a hypothesized potential effect‐modification relationship explored through stratified analyses. Outcomes were assessed at 6 and 12 months. The patient–implant–time hierarchy is shown separately to illustrate the sources of within‐participant correlation and does not represent causal pathways; this dependence was accounted for using mixed‐effects models.

Potential lifestyle and periodontal confounders were also considered. Participants smoking > 10 cigarettes/day and those with active periodontal disease or acute infection at or around the implant site were excluded. No enrolled participant was a smoker; therefore, smoking status had no variability and was not included as a regression covariate. A previous history of periodontitis, as distinct from active periodontal disease, was not modelled as a separate covariate and is acknowledged as a limitation. Clustering arising from two implants per participant and repeated follow‐up measurements was addressed separately using mixed‐effects models.

### 2.9 Statistical Analysis

Data were entered anonymously in Microsoft Excel and analysed using Stata 17. Analyses were guided by the prespecified causal framework (Figure 3). HbA1c category (< 7.0% vs. ≥ 7.0%) and implant diameter (3.3 vs. 4.1 mm) were modelled as fixed effects. For longitudinal outcomes, assessment time (6 and 12 months) was included as a fixed effect. Age, sex and baseline ridge width were prespecified covariates.

Continuous variables were assessed for distributional characteristics and summarized as medians and interquartile ranges (IQRs) because the observed distributions were non‐normal. Descriptive between‐group comparisons used the Mann–Whitney *U* test for continuous variables and chi‐square or Fisher's exact tests for categorical variables.

Because each participant contributed two implants and longitudinal outcomes were measured repeatedly, analyses accounted for within‐participant correlation. Mixed‐effects models were initially evaluated with participant as the clustering unit. Where the participant‐level random‐effect variance was estimated as negligible, inference was retained using participant‐level cluster‐robust standard errors rather than assuming statistical independence of observations. Continuous longitudinal outcomes, including MBL, ISQ, probing depth, plaque index and gingival index, were analysed using linear regression models incorporating the prespecified fixed effects and participant‐level clustering. Binary outcomes, including MBL ≥ 0.5 mm and peri‐implant mucositis, were analysed using logistic regression with participant‐level cluster‐robust standard errors.

For longitudinal outcomes, assessment time was included in the model, and the corresponding treatment‐by‐time interaction was evaluated where appropriate. Model adequacy was assessed using residual‐versus‐fitted and quantile‐normal plots. Normality of the raw outcome variables was not assumed as a prerequisite for linear modelling; rather, the distribution and homoscedasticity of model residuals were examined.

For radiographic MBL calculations, negative values were retained and represented bone gain relative to the reference measurement, whereas positive values represented marginal bone loss. The ordinal Healing Index was analysed descriptively because of its limited distribution across categories, and adjusted modelling was not undertaken. Statistical significance was set at *p* < 0.05, with *p* < 0.001 reported where applicable.

## 3 Results

### 3.1 Patient Allocation/Participant Flow

Figure 4 shows the CONSORT flow chart of the study. A total of 88 participants with 176 implants were enrolled into the study. All participants completed the treatment.

**FIGURE 4:**
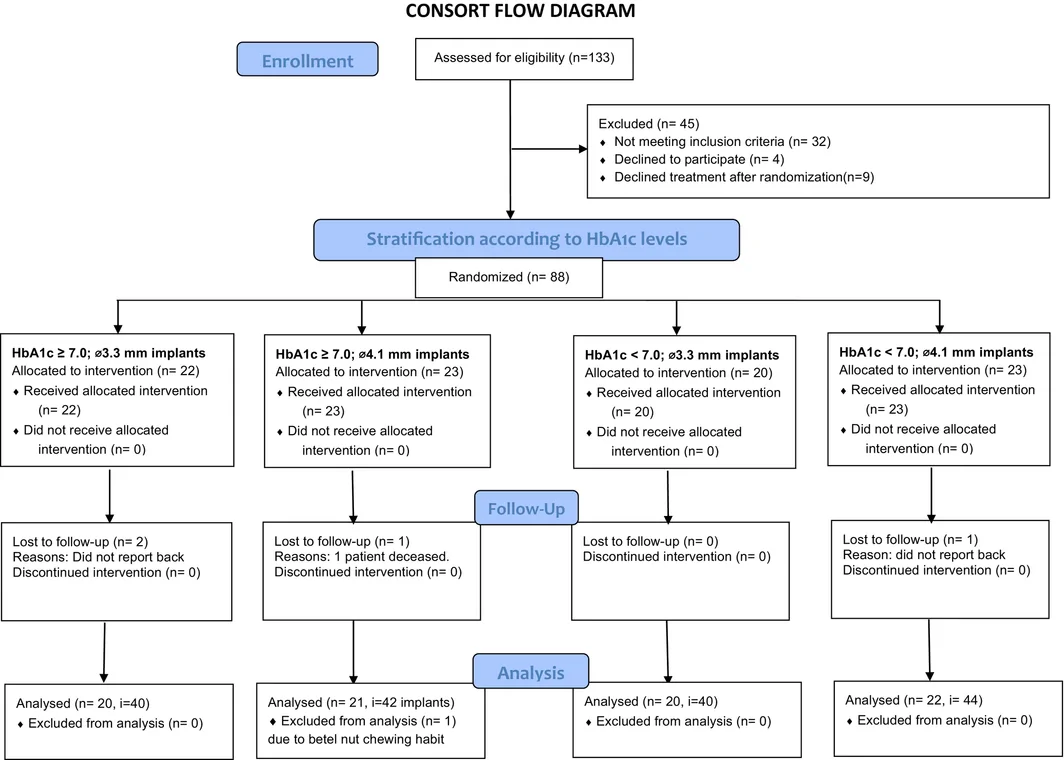
CONSORT flow chart of the study. *i*, number of implants; *n*, number of participants.

After stratification according to HbA1c levels, 45 participants had HbA1c ≥ 7% (exposed), and 43 participants had HbA1c < 7% (unexposed). After randomizing these participants, 42 participants received ⌀3.3 mm (84 implants) (test) and 46 participants received ⌀4.1 mm (92 implants) (control).

The subgroup distribution was as follows: 22 participants received 44 ⌀3.3 mm implants with HbA1c ≥ 7% (test‐exposed); 23 participants received 46 ⌀4.1 mm implants with HbA1c ≥ 7% (control‐exposed); 20 participants received 40 ⌀3.3 mm implants with HbA1c < 7% (test‐unexposed); and 23 participants received 46 ⌀4.1 mm implants with HbA1c < 7% (control‐unexposed).

Five participants were excluded from the outcome analysis: one died from myocardial infarction unrelated to treatment (Group ⌀4.1 mm‐HbA1c ≥ 7%), one was excluded because of resumed heavy betel‐nut chewing (Group ⌀4.1 mm‐HbA1c ≥ 7%), and three were lost to follow‐up (two from Group ⌀3.3 mm‐HbA1c ≥ 7%, and one from Group ⌀4.1 mm‐HbA1c < 7%). Consequently, 83 participants with 166 implants were analysed for outcome measures.

### 3.2 Recruitment

Recruitment of participants began in January 2020 and continued till June 2023. Due to COVID‐19, no recruitment was carried out from March 2020 till December 2020 (first wave), and between March 2021 and August 2021 (second wave). The last subject measured at 12‐month follow up was in October 2024. The trial ended after all participants were recruited.

### 3.3 Baseline Data

A total of 88 participants with 176 implants were considered for the baseline analysis (Table 2).

**TABLE 2:** Baseline demographic and clinical characteristics.

Parameter	Overall		HbA1c ≥ 7%		HbA1c < 7%	
	⌀3.3 mm (*n* = 84 implants in 42 subjects)	⌀4.1 mm (*n* = 92 implants in 46 subjects)	⌀3.3 mm (*n* = 44 implants in 22 subjects)	⌀4.1 mm (*n* = 46 implants in 23 subjects)	⌀3.3 mm (*n* = 40 implants in 20 subjects)	⌀4.1 mm (*n* = 46 implants in 23 subjects)
Age in years median (IQR)	64.5 (53–72)	67 (51–73)	68.5 (62–72)	70 (67–74)	53 (49–66)	62 (48–71)
Sex (M/F)	46/38	40/52	22/22	12/34	24/16	28/18
HbA1c % median (IQR)	7 (6.5–7.6)	6.9 (6.1–7.8)	7.6 (7.1–8.2)	7.8 (7.6–8.8)	6.5 (6.3–6.7)	6.1 (5.9–6.3)
Baseline width in mm median (IQR)	6.3 (6.1–6.7)*	7.2 (6.6–8.1)*	6.47 (6.15–6.72)*	7.2 (6.6–8.2)*	6.17 (6.02–6.4)*	7.07 (6.5–8.1)*
Baseline length in mm median (IQR)	12.3 (10.5–13.7)	12.1 (10.6–13.9)	13.02 (10.8–14.1)	11.9 (10.7–13.1)	11.7 (10.2–13.2)	12.2 (10.6–14.5)
Number of implants 8 mm length	35 (41.6%)	25 (27.1%)	17 (38.6%)	10 (21.7%)	18 (45%)	15 (32.6%)
Number of implants 10 mm length	49 (58.3%)	67 (72.8%)	27 (61.4%)	36 (78.2%)	22 (55%)	31 (67.4%)
2 teeth replaced number (%)	36 (42.8%)	36 (39.1%)	24 (54.5%)	20 (43.5%)	12 (30%)	16 (34.8%)
3 teeth replaced number (%)	48 (57.1%)	56 (60.8%)	20 (45.5%)	26 (56.5%)	28 (70%)	30 (65.2%)

* *p* < 0.01 for comparison between implant‐diameter groups.

There were no significant differences in age, sex, HbA1c levels, baseline bone length or number of teeth replaced between the ⌀3.3 mm and ⌀4.1 mm groups. However, baseline ridge width differed significantly (*p* < 0.01), with participants receiving ⌀3.3 mm implants having a narrower ridge width [median 6.3 mm (IQR 6.1–6.7)] than those receiving ⌀4.1 mm implants [median 7.2 mm (IQR 6.6–8.1)]. Supplement Table 3 reveals the multivariate logistic regression analysis assessing covariates asssociated with implant diameter selection.

### 3.4 Outcome Analysis

After accounting for exclusions and losses to follow‐up, a total of 83 participants with 166 implants were analysed. All implants underwent successful osseointegration and prosthesis delivery (100% implant survival). All implants fulfilled the success criteria according to Buser et al. (1990).

### 3.5 Primary Objective

#### 3.5.1 Marginal Bone Loss (MBL)

At 6 months (T3), the median MBL was higher in the ⌀3.3 mm group than in the ⌀4.1 mm group (0.22 vs. 0.08 mm; cluster‐adjusted *p* = 0.001) (Table 3, Figure 5). At 12 months (T4), the median MBL was 0.27 mm in both groups, with no statistically significant difference after adjustment for patient‐level clustering (*p* = 0.078).

**TABLE 3:** Comparison of patient‐level cluster‐adjusted Implant outcome measures of marginal bone loss, implant stability quotient (isq), peri‐implant indices, frequency of peri‐implant mucositis and healing index between ⌀3.3 mm and ⌀4.1 mm implants.

Parameter	Overall		Adjusted estimate (95% CI)	Corrected *p*
	⌀3.3 mm (*n* = 80)	⌀4.1 mm (*n* = 86)		
*Marginal bone loss* (*MBL*)				
MBL 6‐month mesial	0.24 (0.06–0.58)	0.06 (−0.16 to 0.33)	0.04 (−0.14 to −0.22)	0.659
MBL 6‐month distal	0.17 (0–0.58)	0.09 (−0.24 to 0.5)	0.04 (−0.11 to −0.20)	0.576
MBL 6‐month average	0.22 (0.05–0.51)	0.08 (−0.12 to 0.31)	−0.20 (−0.31 to −0.08)	0.001**
MBL 12‐month mesial	0.34 (0.09–0.73)	0.22 (−0.11 to 0.63)	0.10 (−0.08 to −0.30)	0.282
MBL 12‐month distal	0.17 (0.02–0.73)	0.33 (−0.08 to 0.66)	0.02 (−0.17 to −0.20)	0.869
MBL 12‐month average	0.27 (0.07–0.68)	0.27 (−0.04 to 0.54)	−0.13 (−0.28 to −0.01)	0.078
*MBL 6 months*				
< 0.5 mm	58 (72.5%)	77 (89.5%)	−1.63 (−2.66 to −0.60)	0.002**
> = 0.5 mm	22 (27.5%)	9 (10.5%)		
*MBL 12 months*				
< 0.5 mm	52 (65%)	61 (70.9%)	−0.07 (−0.87 to 0.72)	0.852
> = 0.5 mm	28 (35%)	25 (29.1%)		
*Implant stability quotient* (*ISQ*)				
ISQ average T0–primary	69.8 (64.8–75)	74.6 (69.8–78.8)	4.37 (1.53 to 7.21)	0.003**
ISQ average T2–3 months	76.1 (74.1–79.4)	79.6 (76–81.8)	3.15 (1.47 to 4.83)	0.0001***
Difference	6.75 (2.41–11.2)	4.25 (0.5–9.16)		
*Clinical peri‐implant Indices*				
Plaque Index average 6 months	0.25 (0–0.5)	0.25 (0–0.5)	0.02 (−0.09 to −0.14)	0.731
Gingival Index average 6 months	0 (0–0.25)	0 (0–0.25)	−0.03 (−0.09 to −0.02)	0.265
Probing depth average 6 months	2.5 (2.25–3)	2.25 (2–2.5)	−0.17 (−0.28 to −0.06)	0.002**
Plaque Index average 12 months	0.25 (0–0.5)	0.5 (0–0.5)	0.08 (−0.06 to 0.23)	0.265
Gingival Index average 12 months	0 (0–0.25)	0 (0–0.5)	0.03 (−0.05 to 0.12)	0.447
Probing depth average 12 months	2.5 (2.25–3)	2.25 (2–2.5)	−0.18 (−0.31 to −0.05)	0.005**
*Peri‐implant mucositis* (*any one site around an implant*)				
At 6‐month post‐prosthesis delivery				
Absent	52 (65%)	60 (69.8%)	0.03 (−0.66 to 0.72)	0.928
Present	28 (35%)	26 (30.2%)		
At 12‐month post‐prosthesis delivery				
Absent	49 (61.3%)	44 (51.2%)	−0.09 (−0.89 to 0.71)	0.823
Present	31 (38.7%)	42 (48.8%)		
Healing Index				
Healing Index 14 days	5 (4–5)	4 (4–5)		0.005**
Healing Index 3 months	5 (5–5)	5 (5–5)		0.530

*Note:* Continuous variables are presented as median (interquartile range) and categorical variables as frequency (percentage). All adjusted estimates, 95% CIs, and *p*‐values are derived from mixed‐effects regression models utilizing patient‐level random intercepts to control for intra‐subject clustering (two implants per patient). Continuous estimates are *β* coefficients from linear mixed‐effects regression; binary estimates are ORs from mixed‐effects logistic regression with patient‐level random intercepts.

Abbreviations: CI, confidence interval; GI, Gingival Index; HI, Healing Index by Landry and Howley (1988); ISQ, implant stability quotient; MBL, marginal bone level changes; PD, probing depth.

*** *p* < 0.05.

** *p* < 0.01.

**FIGURE 5:**
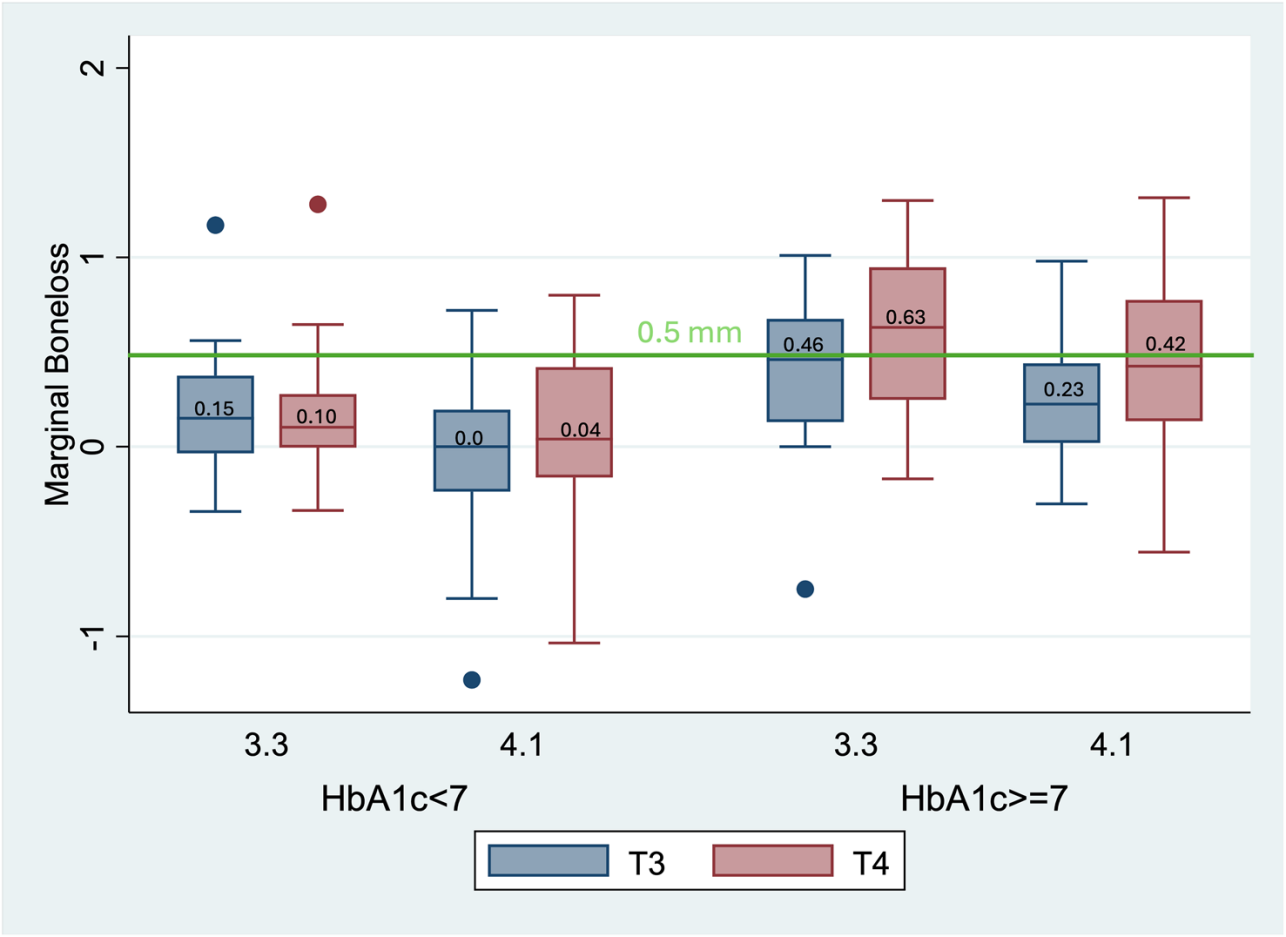
Box plot displaying the marginal bone changes in ⌀3.3 mm and ⌀4.1 mm implants at the 6‐month (T3) and 12‐month (T4) time points in the HbA1c < 7% and HbA1c ≥ 7% sub‐groups. Positive values indicate bone loss and negative values bone gain.

Table 4 reveals subgroup analysis of ⌀3.3 mm and ⌀4.1 mm implants depending on HbA1c levels. Among participants with HbA1c ≥ 7%, the ⌀3.3 mm group had the highest median 12‐month MBL (0.63 mm), compared with 0.43 mm in the ⌀4.1 mm group. Group HbA1c < 7% showed better outcomes. At 12 months, group HbA1c < 7% showed no significant differences in the marginal bone loss between ⌀3.3 mm and ⌀4.1 mm implants, and this was superior as compared to the bone loss seen in group HbA1c ≥ 7% for both diameters.

**TABLE 4:** Comparison of patient‐level cluster‐adjusted implant outcome measures between ⌀3.3 mm and ⌀4.1 mm implants stratified according to HbA1c ≥ 7% and HbA1c < 7%.

Parameter	HbA1c ≥ 7				HbA1c < 7			
	⌀3.3 mm	⌀4.1 mm	Adjusted estimate (95% CI)	*p*	⌀3.3 mm	⌀4.1 mm	Adjusted estimate (95% CI)	*p*
	(*n* = 40)	(*n* = 42)			(*n* = 40)	(*n* = 44)		
*Marginal bone loss*								
MBL 6 months mesial	0.3 (0.11–0.68)	0.13 (−0.09 to 0.5)	0.22 (−0.06 to 0.50)	0.131	0.15 (−0.03 to 0.44)	0.01 (−0.31 to 0.25)	−0.09 (−0.33 to 0.15)	0.466
MBL 6 months distal	0.29 (0.02–0.8)	0.37 (0–0.59)	−0.09 (−0.32 to 0.13)	0.408	0.13 (−0.04 to 0.19)	−0.05 (−0.37 to 0.15)	0.19 (−0.28 to 0.42)	0.124
MBL 6 months average	0.5 (0.12–0.7)	0.22 (0.01–0.44)	−0.25 (−0.44 to −0.07)	0.005**	0.15 (−0.04 to 0.37)	−0.05 (−0.23 to 0.19)	−0.17 (−0.33 to −0.01)	0.031*
MBL 12 months mesial	0.64 (0.3–1.01)	0.36 (0–0.82)	0.21 (−0.07 to 0.50)	0.145	0.13 (−0.04 to 0.45)	0.01 (−0.24 to 0.53)	0.06 (−0.22 to 0.34)	0.667
MBL 12 months distal	0.7 (0.22–1.05)	0.58 (0.31–0.8)	0.06 (−0.19 to 0.31)	0.628	0.06 (0–0.14)	0.01 (−0.13 to 0.36)	−0.02 (−0.32 to 0.27)	0.877
MBL 12 months average	0.63 (0.24–0.94)	0.43 (0.13–0.78)	−0.12 (−0.33 to 0.08)	0.241	0.10 (0–0.27)	0.04 (−0.16 to 0.42)	−0.17 (−0.39 to 0.04)	0.126
*MBL 6 months*								
< 0.5 mm	22 (55%)	34 (80.9%)	−2.04 (−5.0 to 0.92)	0.177	36 (90%)	43 (97.7%)	−1.54 (−2.68 to 0.42)	0.007**
≥ 0.5 mm	18 (45%)	8 (19.1%)			4 (10%)	1 (2.3%)		
*MBL 12 months*								
< 0.5 mm	16 (40%)	24 (57.2%)	−0.32 (−1.80 to 1.14)	0.663	36 (90%)	37 (84.1%)	0.01 (−1.44 to 1.47)	0.988
≥ 0.5 mm	24 (60%)	18 (42.8%)			4 (10%)	7 (15.9%)		
*ISQ measurements*								
ISQ average T0–primary	70 (66.3–74.1)	77.3 (72.5–80.1)	3.76 (−0.21 to 7.75)	0.064	68.9 (63.7–75.3)	71.7 (69–76.1)	5.47 (1.24–9.70)	0.011*
ISQ average T2–3 months	74.7 (71.5–76.7)	79.8 (76–82)	1.65 (−0.30 to 3.61)	0.097	78.5 (75.6–80)	79.6 (76.5–81)	4.39 (1.59–7.19)	0.002**
*Clinical peri‐implant indices*								
Plaque Index average 6 months	0.25 (0–0.5)	0.25 (0–0.5)	0.02 (−0.14 to 0.19)	0.796	0.25 (0–0.5)	0.25 (0–0.5)	0.07 (−0.10 to 0.24)	0.431
Gingival Index average 6 months	0 (0–0.25)	0.5 (0–0.25)	−0.03 (−0.11 to 0.05)	0.455	0 (0–0.25)	(0–0)	−0.02 (−0.12 to 0.06)	0.602
Probing depth average 6 months	2.5 (2.4–3)	2.25 (2.25–2.5)	−0.21 (−0.39 to −0.03)	0.017**	2.5 (2.25–3)	2.25 (2–2.5)	−0.12 (−0.29 to 0.03)	0.129
Plaque Index average 12 months	0.25 (0–0.75)	0.5 (0–0.1)	0.15 (−0.05 to 0.36)	0.154	0.13 (0–0.5)	0.38 (0.13–0.5)	0.0 (−0.15 to 0.28)	0.570
Gingival Index average 12 months	0 (0–0.5)	0.25 (0–0.5)	0.04 (−0.07 to 0.16)	0.04*	0 (0–0.25)	0 (0–0.25)	0.02 (−0.11 to 0.15)	0.735
Probing depth average 12 months	2.5 (2.25–3)	2.25 (2–2.5)	−0.18 (−0.37 to 0.01)	0.059	2.5 (2.25–3)	2.25 (2–2.5)	−0.15 (−0.36 to 0.04)	0.124
*Peri implant mucositis* (*any one site around an implant*)								
Absent	24 (60%)	26 (62%)	−2.03 (−4.95 to 0.89)	0.174	28 (70%)	34 (77.3%)	−1.55 (−2.68 to 0.42)	0.007**
Present	16 (40%)	16 (38%)	—		12 (30%)	10 (22.7%)		
*At 12 month*								
Absent	21 (52.5%)	17 (40.5%)	0.36 (−1.21 to 1.94)	0.655	28 (70%)	27 (61.4%)	−0.49 (−1.49 to 0.49)	0.325
Present	19 (47.5%)	25 (59.5%)			12 (30%)	17 (38.6%)		
*Healing Index*								
Healing Index 14 days	4.5 (4–5)	4 (4–4)		0.091	5 (5–5)	4 (4–5)		0.017*
Healing Index 3 months	5 (5–5)	5 (5–5)		0.512	5 (5–5)	5 (5–5)		1.000

*Note:* Continuous parameters are reported as median (interquartile range) and categorical parameters as frequency (percentage). Adjusted estimates show the effect of 4.1 mm diameter versus 3.3 mm reference. Models utilized mixed‐effects linear regression for continuous data and mixed‐effects logistic regression for categorical data, applying patient‐level random intercepts to account for clustering. Continuous estimates are *β* coefficients from linear mixed‐effects regression; binary estimates are ORs from mixed‐effects logistic regression with patient‐level random intercepts.

Abbreviations: CI, confidence interval; GI, Gingival Index; HI, Healing Index by Landry and Howley (1988); ISQ, implant stability quotient; MBL, marginal bone loss; PD, probing depth; PI, Plaque Index.

* *p* < 0.05.

** *p* < 0.01.

#### 3.5.2 Clinically Relevant MBL ≥ 0.5 mm

After 1 year of prosthetic loading, a higher proportion of MBL ≥ 0.5 mm was observed in the ⌀3.3 mm implant group (*n* = 28, 35%) compared to the ⌀4.1 mm group (*n* = 25, 29%). However, this difference was not statistically significant (*p* = 0.852) (Tables 3 and 4, Figure 5). Irrespective of implant diameter, a greater proportion of implants in the HbA1c ≥ 7% group had MBL ≥ 0.5 mm at 12 months (42/82, 51.2%) compared with the HbA1c < 7% group (11/84, 13.1%; *p* < 0.001) (Table 5). In addition, a positive correlation was seen between HbA1c and MBL for both ⌀3.3 mm (Spearman's 𝞺=0.3112, *p* = 0.005) and ⌀4.1 mm (Spearman's 𝞺 = 0.4069, *p* < 0.001) implants (Figure 6).

**TABLE 5:** Logistic regression model: Risk factors for marginal bone loss ≥ 0.5 mm: Impact of implant diameter and glycaemic control.

Parameter	MBL_Avg_T3				MBL_Avg_T4			
	Unadjusted		Adjusted		Unadjusted		Adjusted	
	Odds	*p*	Odds	*p*	Odds	*p*	Odds	*p*
*Implants*								
⌀3.3								
⌀4.1	0.31	0.006**	0.272	0.005**	0.76	0.413	0.738	0.407
*HbA1c category*								
HbA1c < 7								
HbA1c ≥ 7	7.33	< 0.001***	8.00	< 0.001***	6.96	< 0.001***	7.00	< 0.001***

** Significant at 1% level.*** Significant at 0.1% level.

**FIGURE 6:**
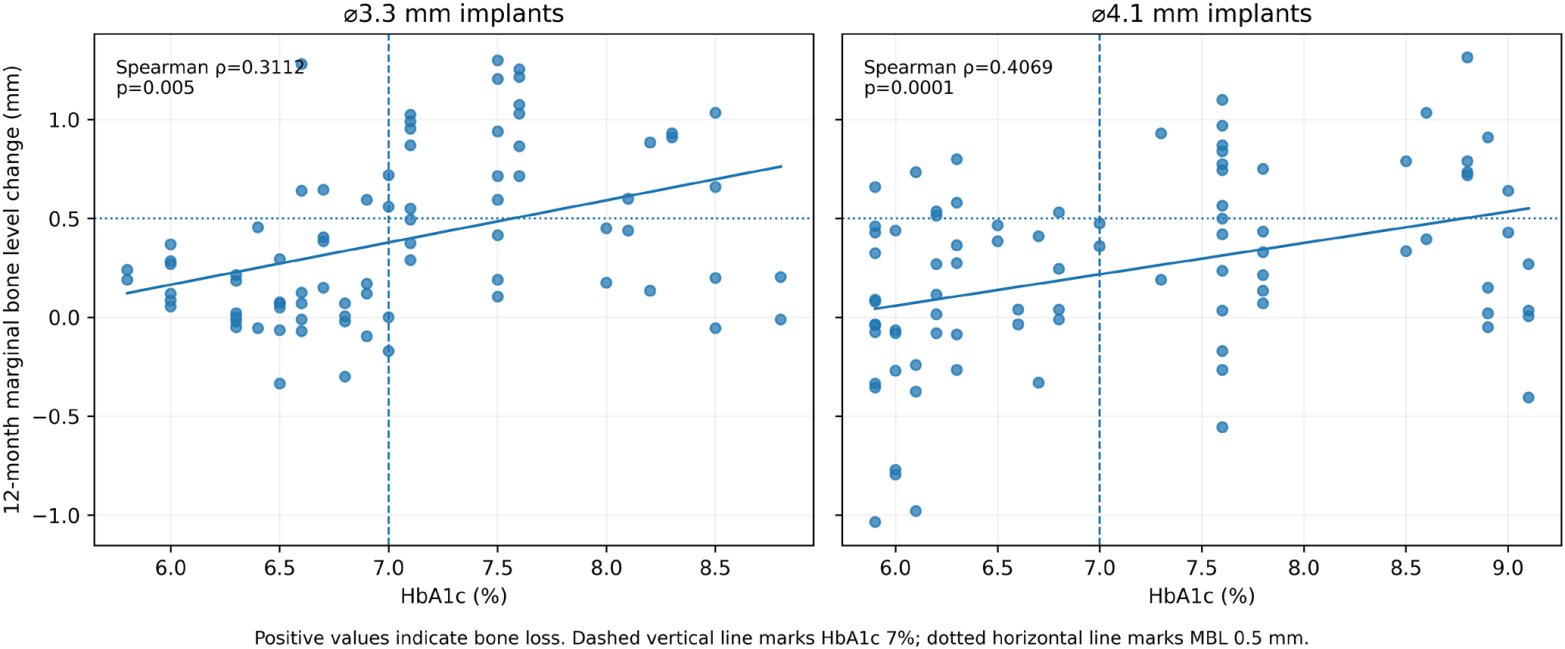
Correlation between HbA1c (%) (*X*‐axis) and marginal bone loss (mm) (*Y*‐axis) in relation to implant diameter (⌀3.3 mm or ⌀4.1 mm). For 3.3 mm: Rho = 0.3112; *p* = 0.005; For 4.1 mm: Rho = 0.4069; *p* = 0.0001.

Logistic regression was performed to assess factors associated with MBL ≥ 0.5 mm, with implant diameter and HbA1c category included as predictors (Table 5). It revealed that participants with HbA1c ≥ 7% have 6.96 to 8.00 times higher odds of experiencing MBL ≥ 0.5 mm than HbA1c < 7%. This association remained statistically significant after adjustment (OR 7.00, *p* < 0.001). Although implant diameter impacts marginal bone loss, diameter alone is not a significant predictor of high‐risk ‘odds’ (*p* > 0.40). Table S3 shows baseline and supplement table S4 shows outcome parameters when pooling data of HbA1c ≥ 7% versus HbA1c < 7%.

### 3.6 Secondary Objective

#### 3.6.1 Implant Stability

Twenty‐seven implants (in 18 participants) had primary ISQ < 65 and underwent submerged healing. At 3 months, all implants had ISQ > 65 and were loaded with a prosthesis. The ⌀4.1 mm group demonstrated significantly higher primary and secondary ISQ values than the ⌀3.3 mm group (*p* = 0.003 and *p* < 0.001, respectively) (Tables 3 and 4, Figure S1).

#### 3.6.2 Peri‐Implant Clinical Outcomes

Probing depth was modestly greater around ⌀3.3 mm implants than around ⌀4.1 mm implants at both 6 months (*p* = 0.002) and 12 months (*p* = 0.005), although values remained within clinically healthy limits. No implant had a site with PD ≥ 5 mm (Tables 3 and 4).

No implants had peri‐implantitis at any time point. The frequency of peri‐implant mucositis has been elaborated in Tables 3 and 4. These differences were not statistically significant between any of the groups.

#### 3.6.3 Healing Index (HI)

At 2 weeks post‐implant placement, 42 participants scored excellent (HI5), 33 scored very good (HI4), 5 scored good (HI3), 2 scored poor (HI2) and 1 scored very poor (HI1) healing. The 2 participants with poor and 1 patient with very poor were from ⌀4.1‐HbA1c ≥ 7 group. At 3 months post‐implant placement, all participants had excellent healing (HI5) except 2 participants, from ⌀4.1‐HbA1c ≥ 7% group, who had very good healing (HI4). At 14 days, the Healing Index was significantly higher in the ⌀3.3 mm group than in the ⌀4.1 mm group (*p* = 0.005); by 3 months, Healing Index scores were similar between groups (*p* = 0.530) (Tables 3 and 4).

#### 3.6.4 Patient Reported Outcome Measures (PROMs)

Table 6 elaborates results of OHIP‐14. Out of the 14 questions in the OHIP‐14 questionnaire, questions 1, 3, 6, 10, 11, 12 and 14 were scored as 0 by all participants for both time points T0 and T4. This indicated that these questions were not applicable to this group of participants.

**TABLE 6:** Comparison of OHIP‐14 and DSI scores (at baseline and at 12 months following prosthesis delivery) between ⌀3.3 and ⌀4.1 mm implants, and further sub grouped as per HbA1c ≥ 7% and HbA1c < 7%.

OHIP	⌀3.3			⌀4.1			Overall (⌀3.3 vs. ⌀4.11)	HbA1c ≥ 7.0% (⌀3.3 vs. ⌀4.1)	HbA1c < 7.0% (⌀3.3 vs. ⌀4.1)
	Baseline	12 months	*p*	Baseline	12 months	*p*	*p*	*p*	*p*
1 Functional limitation_1	0 (0–0)	0 (0–0)	—	0 (0–0)	0 (0–0)	—	—	—	—
2 Functional limitiation_2	0 (0–0)	0 (0–0)	0.011^s^ *	0.16 (0.37)	0 (0–0)	0.011^s^ *	—	0.6262^M^	0.8233^M^
3 Physical pain_1	0 (0–0)	0 (0–0)	—	0 (0–0)	0 (0–0)	—	—	—	—
4 Physical pain_2	3 (3–3)	0 (0–0)	< 0.001^s^ ***	3 (2–3)	0 (0–0)	< 0.001^s^ ***	0.649^M^	0.829^M^	0.684^M^
5 Psychological discomfort_1	1 (1–1)	0 (0–0)	< 0.001^s^ ***	1 (1–1)	0 (0–0)	< 0.001^s^ ***	0.953^M^	0.776^M^	0.707^M^
6 Psychological discomfort_2	0 (0–0)	0 (0–0)	—	0 (0–0)	0 (0–0)	—	—	—	—
7 Physical disability_1	3 (3–3)	0 (0–0)	< 0.001^s^ ***	3 (2–3)	0 (0–0)	< 0.001^s^ ***	0.695^M^	0.829^M^	0.747^M^
8 Physical disability_2	0 (0–1)	0 (0–0)	< 0.001^S^ ***	0 (0–1)	0 (0–0)	< 0.001^S^ ***	0.528^M^	0.688^M^	0.837^M^
9 Psychological disability_1	0 (0–0)	0 (0–0)	0.240^S^	0 (0–0)	0 (0–0)	0.2410^S^	—	—	—
10 Psychological disability_2	0 (0–0)	0 (0–0)	—	0 (0–0)	0 (0–0)	—	—	—	—
11 Social disability_1	0 (0–0)	0 (0–0)	—	0 (0–0)	0 (0–0)	—	—	—	—
12 Social disability_2	0 (0–0)	0 (0–0)	—	0 (0–0)	0 (0–0)	—	—	—	—
13 Handicap_1	0 (0–0)	0 (0–0)	1.0000^S^	0 (0–0)	0 (0–0)	0.0551^S^	0.246^M^	—	0.278^M^
14 Handicap_2	0 (0–0)	0 (0–0)	—	0 (0–0)	0 (0–0)	—	—	—	—
Chewing Ability	8 (8–9)	10 (10–10)	< 0.001^S^ ***	8 (8–9)	10 (10–10)	< 0.001^S^ ***	0.928^M^	0.861^M^	0.760^M^

*Note:* Scoring for OHIP‐14 is on a Likert Scale from 0 to 4 for each question, 0 = never, 1 = 4 = very often; Chewing Ability was assessed using a numerical rating scale from 0 to 10, with higher scores indicating better chewing ability.

Abbreviations: *M*, Mann–Whitney *U* test; s, sign rank.

* Significant at 5% level (*p* < 0.05).

*** Significant at 0.1% level (*p* < 0.001).

For questions 2, 4, 5, 7, 8 and 13 there was a statistically significant improvement in the scores between baseline and 12 months post‐prosthesis. The maximum improvement was seen for Question 7 related to physical disability (Has your diet been unsatisfactory because of problems with your teeth, disability mouth or dentures?) and for Question 4 related to physical pain (Have you found it uncomfortable to eat any foods because of problems with your teeth, mouth or dentures?). There was a statistically significant improvement in the numerical rating scale for chewing ability between baseline and 12 months post‐prosthesis.

There were no statistically significant differences in PROMs or chewing ability between the ⌀3.3 mm and ⌀4.1 mm groups, either overall or within the HbA1c ≥ 7% and HbA1c < 7% subgroups.

### 3.7 Adverse Events, Technical and Prosthetic Complications

Two participants who underwent transmucosal healing (both ⌀3.3 mm‐HbA1c ≥ 7%), presented with dislodged healing abutments and closed wounds when arriving for the prosthetic phase. Healing abutments were reinstalled, and prosthetic phase began 2 weeks later. Two participants had screw loosening at 12 months post‐prosthesis (1 from ⌀3.3 mm‐HbA1c < 7% and 1 from ⌀4.1 mm‐HbA1 < 7%), and the occlusal screws were tightened. One patient presented with chipping of the porcelain 9 months following prosthesis delivery (⌀3.3 mm‐HbA1c ≥ 7%) and 2 participants presented with debonding of the fixed prosthesis from the abutment 8 and 10 months following prosthesis delivery (⌀4.1 mm‐HbA1c < 7% and ⌀4.1 mm‐HbA1c ≥ 7%). One of them had accidentally ingested the restoration, and it passed out in stools without additional intervention, 5 days following ingestion.

Beyond the above‐mentioned complications, no participants were harmed. No device‐related adverse events were reported during the trial.

### 3.8 Post Hoc Power Analysis

A post hoc power analysis was performed in response to a reviewer's request using *α* = 0.05 and 80% power. Based on the observed sample size, the analysis indicated adequate power to detect a mean difference of 0.16 mm in marginal bone loss. A sensitivity analysis incorporating an ICC of 0.50 was also performed to account for within‐participant clustering.

## 4 Discussion

The central finding of this stratified randomized clinical trial is that, at 1 year after prosthetic loading, ⌀3.3‐mm hydrophilic Ti‐Zr implants did not demonstrate a statistically significant difference in MBL compared with ⌀4.1‐mm implants. Implant survival was 100% in the analysed cohort, with no peri‐implantitis observed at 1‐year post‐prosthesis delivery. The clinically more striking signal was metabolic: HbA1c ≥ 7% was associated with substantially greater MBL and approximately sevenfold higher odds of MBL ≥ 0.5 mm. The wider implants nevertheless demonstrated consistently greater early mechanical stability, while early soft‐tissue healing favoured the narrower implants and these differences largely resolved by 3 months.

### 4.1 Contribution of Implant Diameter

The lower 6‐month MBL and higher ISQ values observed with ⌀4.1‐mm implants are biologically coherent (Kumar et al. 2017; Qiu et al. 2024; Wang et al. 2023). A wider implant provides a larger bone–implant interface and may distribute functional forces over a broader crestal area (Ding et al. 2009; Zweers et al. 2015). The transient 6‐month difference suggests that implant diameter may influence the early remodelling environment without necessarily determining the 1‐year biological endpoint. The absence of a significant 12‐month difference is particularly relevant clinically because narrow implants may permit treatment of sites in which a wider implant would require augmentation.

The present results should not be described as proof of equivalence or non‐inferiority. The study was powered around a conventional superiority difference and did not use a formal equivalence or non‐inferiority margin. The appropriate interpretation is that no statistically significant difference was detected at 12 months in this selected cohort.

### 4.2 Glycaemic Status Is the Stronger Biological Signal

The magnitude and consistency of the HbA1c association distinguish it from the diameter comparison. Participants were stratified using a pre‐specified HbA1c threshold (HbA1c ≥ 7% vs. HbA1c < 7%), aligned with American Diabetes Association targets for non‐pregnant adults (Wagner et al. 2022). Given variability in definitions of ‘uncontrolled’ diabetes in the dental literature (Al‐Sowygh et al. 2018; Eskow and Oates 2017), outcomes are presented relative to this cut‐off. Throughout the study, plaque and gingival indices remained consistently low across the groups, suggesting that inadequate oral hygiene was unlikely to be a major contributor to the observed bone loss. At 12 months, more than half of implants in participants with HbA1c ≥ 7% crossed the 0.5‐mm MBL threshold compared with approximately one in eight implants among participants with HbA1c < 7%. The association remained strong after adjustment, and continuous HbA1c correlated positively with MBL in both diameter groups.

These findings are compatible with the biological consequences of chronic hyperglycaemia, including altered inflammatory signalling, impaired tissue repair and disturbed bone remodelling (Javed and Romanos 2009; Jiang et al. 2021). More recently, (Trullenque‐Eriksson et al. 2025), in a large Swedish register‐based cohort, reported an increased risk of peri‐implantitis among individuals with diabetes and highlighted glycaemic control as a potentially important factor in the relationship between diabetes and peri‐implant disease. Their findings provide contemporary population‐level evidence that complements the present results, although direct comparison should be made cautiously because their primary outcome was peri‐implantitis rather than marginal bone loss. The distinction is important: this study demonstrates an association between glycaemic status and early bone change, not a causal effect of hyperglycaemia on peri‐implantitis.

The clinical implication is therefore one of risk stratification rather than exclusion. All implants survived, all ultimately achieved loading, and complete or near‐complete healing was observed by 3 months. Elevated HbA1c should not be interpreted from these data as an absolute contraindication to implant therapy. Rather, it identifies a population in whom glycaemic optimization, meticulous maintenance and closer early monitoring may be particularly valuable.

### 4.3 The Interaction Between Anatomy and Treatment Choice

Ridge width is the most important structural limitation of the diameter comparison. The protocol intentionally allowed ⌀3.3‐mm implants to be placed in ridges as narrow as 5.3 mm, whereas ⌀4.1‐mm implants required augmentation below approximately 6.1 mm. Consequently, implant diameter was not independent of local anatomy. The supplementary allocation analysis confirms that ridge width was strongly associated with the diameter actually received. Several participants, particularly those with HbA1c ≥ 7%, declined augmentation, resulting in limited augmentation in the ⌀3.3 mm group.

This is not merely a selection bias resulting in a statistical flaw. It reflects the clinical decision and patient preferences that make narrow implants useful in the first place. A trial that required every participant to have enough bone for either diameter would offer a cleaner randomized comparison but would exclude precisely the patients for whom a narrow implant is most clinically valuable. This study therefore has high pragmatic relevance, but the results should be interpreted as applying to the treatment pathway studied rather than as a pure diameter‐only biological experiment.

### 4.4 Mechanical Stability and Biological Healing: Distinct Dimensions of Implant Performance

The higher primary and 3‐month ISQ values observed for ⌀4.1 mm implants indicate a clear mechanical advantage. However, ⌀3.3 mm implants achieved clinically acceptable stability by 3 months, and no statistically significant difference in 12‐month MBL was detected between implant diameters. In contrast, early wound healing was more favourable with ⌀3.3 mm implants, with poorer healing outcomes concentrated primarily in the ⌀4.1 mm/HbA1c ≥ 7% subgroup. Collectively, these findings suggest that primary stability, soft‐tissue healing and marginal bone preservation represent related but distinct dimensions of implant performance.

### 4.5 Patient‐Centred Outcomes

Posterior tooth loss compromises masticatory function (Asher et al. 2025) and may promote reliance on soft diets that are low in fibre and high in carbohydrates, thereby worsening glycaemic control (Bayram and Eskan 2023; Da et al. 2022). Improved mastication and dietary modification have been associated with better glycaemic control in people with diabetes (Riente et al. 2024). In mandibular distal extension situations, the restorability of dentition is either by a tooth‐tissue supported removable prosthesis or with an implant‐supported prosthesis (Herrera et al. 2022). Implant‐supported prostheses offer a stable solution for restoring mastication (Bandiaky et al. 2022; Herrera et al. 2022), particularly when patients decline removable options. The improvement in chewing ability and selected OHIP‐14 domains in this study demonstrates that the intervention produced a meaningful functional benefit beyond radiographic and implant‐level endpoints. Importantly, this improvement was not dependent on implant diameter or HbA1c category. Whether improved mastication subsequently modifies diet or glycaemic control cannot be determined from this study and should not be inferred.

### 4.6 Strengths

The study has several strengths: HbA1c stratification was incorporated before randomization; implant diameter was randomized within strata; the study addressed a clinically relevant posterior indication; two implants per participant and repeated observations were explicitly accounted for statistically; outcomes spanned radiographic, mechanical, soft‐tissue, healing and patient‐reported domains; and a DAG provided a transparent rationale for covariate adjustment. The combination of a clinically meaningful anatomical indication and a metabolically characterized population makes the study particularly relevant to contemporary implant practice.

Evidence on implant outcomes in individuals with HbA1c ≥ 7% remains limited, and most published studies have evaluated single‐tooth implants in small cohorts (Al‐Shibani et al. 2019; Alsahhaf et al. 2019; Cabrera‐Dominguez et al. 2020; Diehl et al. 2024; Latimer et al. 2022). This study extends this evidence by assessing mandibular distal‐extension rehabilitation in a larger sample using both ⌀3.3 mm and ⌀4.1 mm implants.

The favourable outcomes observed may be attributable to careful patient selection, comprehensive patient education, adherence to minimally invasive protocols and use of well‐documented implant systems. Despite the challenges posed by the COVID‐19 pandemic, the cohort demonstrated high compliance, with participants remaining well informed and attending scheduled follow‐up visits throughout the study. Participants received instruction on oral hygiene and the importance of post‐operative maintenance. Such education‐based interventions have been shown to reduce complications in individuals with diabetes (Farhat 2023; Sathyanath et al. 2022).

### 4.7 Limitations

The primary outcome analysis was per protocol, and participants who declined augmentation after allocation to 4.1‐mm implants were excluded from the outcome analysis. This may introduce selection related to anatomy, treatment preference or unmeasured characteristics. Allocation and treatment were performed by the operating clinician, and neither participants nor outcome assessors were blinded. Radiographic readings were reached by consensus between two examiners rather than through an independent reliability analysis.

Follow‐up was limited to 1 year after prosthesis delivery and although the first year after prosthesis delivery is clinically important—particularly in people with diabetes who may be at risk of early implant loss (Fiorellini et al. 2000), longer observation is required to evaluate peri‐implantitis, mechanical failure and other late complications. There was no non‐diabetic comparator group (HbA1c < 5.8), so the study cannot quantify how either HbA1c stratum compares with metabolically healthy adults. Healing protocol was determined by primary stability, meaning that submerged and transmucosal healing were not uniform. Finally, the high level of patient education and structured follow‐up may limit generalizability to routine practice. Such adherence may be more difficult to achieve in routine practice, particularly among individuals with diabetes who face socioeconomic barriers or competing medical burdens (Sathyanath et al. 2022). Accordingly, although the present findings suggest that either implant diameter may be safe and effective in individuals with HbA1c ≥ 7%, translation to everyday clinical settings without sustained emphasis on maintenance and glycaemic optimization may increase the risk of treatment failure.

### 4.8 Implications for Future Research

The most informative next step would be a longer‐term, adequately powered randomized trial restricted to an anatomical range in which both implant diameters can be placed without augmentation, and the effect of these interventions on participant diets and glycaemic control. Such a design could test whether the early mechanical advantage of wider implants produces a durable biological benefit and could prospectively improve HbA1c levels as a continuous exposure. Longer follow‐up should include peri‐implantitis, prosthetic complications and maintenance burden as clinically meaningful endpoints.

## 5 Conclusion

In this stratified randomized clinical trial, ⌀3.3‐mm hydrophilic Ti‐Zr implants supporting splinted fixed dental prostheses in mandibular distal‐extension situations showed no statistically significant difference in 12‐month MBL compared with ⌀4.1‐mm implants. The wider implants provided greater early mechanical stability, whereas early healing favoured the narrower implants and largely normalized by 3 months. The strongest biological signal was glycaemic status: HbA1c ≥ 7% was associated with greater MBL and approximately sevenfold higher odds of MBL ≥ 0.5 mm. Either diameter implant supported prosthetic rehabilitation substantially improved chewing ability. These findings support individualized treatment planning that integrates ridge anatomy, implant dimensions and metabolic status, while recognizing that longer‐term studies are required to establish durability.

## Author Contributions

**Supriya Ebenezer:** conceptualization, methodology, investigation, validation, data curation, supervision, resources, project administration, formal analysis, visualization, writing – review and editing, writing – original draft, software, funding acquisition. **Alampalli V. Ramesh:** methodology, data curation, validation, project administration, resources, writing – original draft, writing – review and editing, investigation. **Chitradevi Ramanathan:** data curation, software, formal analysis, visualization, writing – review and editing. **Sreelakshmi Viswanath:** investigation, validation, data curation, writing – review and editing, writing – original draft. **Vinay V. Kumar:** conceptualization, methodology, software, data curation, investigation, validation, formal analysis, supervision, funding acquisition, visualization, project administration, resources, writing – original draft, writing – review and editing.

## Funding

This work was funded by International Team for Implantology, LG_2017_1250.

## Conflicts of Interest

The authors declare no conflicts of interest. S.E. received speaker fees/honoraria from Straumann Group, ITI and Geistlich Pharma AG, Switzerland, and travel support from ITI. S.V., R.C. and A.V.R. have no conflicts of interest to disclose. V.V.K. has received a grant from Oral Reconstruction Foundation, Basel for an investigator initiated study; received speaker fees/honoraria from Straumann Group, Camlog Biotechnologies GmbH, Basel Switzerland; received support for attending meetings from International Team for Implantology and Oral Reconstruction Foundation. In addition to this study, V.V.K. has received materials from Camlog Biotechnologies GmbH, Basel Switzerland.

## Supporting information


**Figure S1:** Box plot displaying the ISQ changes in ⌀3.3 mm and ⌀4.1 mm implants at the baseline (T0) and 3‐month post‐implant placement (T2) time points in the HbA1c < 7% and HbA1c7% sub‐groups.

**Table S1:** The PICOT for the study.

**Table S2:** Multivariate logistic regression analysis assessing covariates associated with the groups ⌀3.3 mm and ⌀4.1 mm diameter implants.

**Table S3:** Multivariate logistic regression analysis assessing covariates associated with the glycaemic control (HbA1c < 7 vs. HbA1c ≥ 7%).

**Table S4:** Subgroup analysis comparing participants with HbA1c ≥ 7% and HbA1c < 7% for primary and secondary outcome measures.

## Data Availability

The data that support the findings of this study are available on request from the corresponding author. The data are not publicly available due to privacy or ethical restrictions.
