# Supplementary material for: Stratified Randomized Controlled Trial of ∅3.3 mm Versus ∅4.1 mm Hydrophilic Titanium‐Zirconium Implants Supporting Posterior Mandibular Fixed Prostheses in Hyperglycaemic Subjects
Source: Clin Oral Implants Res. 2026 Sep 24;37(11):1328–49. doi: 10.1111/clr.70175 (PMC13743795; doi:10.1111/clr.70175)
Supplement: Supplementary file 1 [file CLR-37-1328-s001.docx]

**Supplement Table 2: Multivariable logistic regression analysis assessing covariates associated with implant diameter selection (⌀3.3 mm vs. ⌀4.1 mm).**

| **Parameter** | **Univariate** |  | **Multivariate** |  |
| --- | --- | --- | --- | --- |
|  | **Odds(95%CI)** | **P value** | **odds(95%CI)** | **P value** |
| **Age(per year Increase)** | 1.01(0.98-1.03) | 0.458 | 1.02(0.98-1.05) | 0.245 |
| **Sex(Female Vs Male)** | 1.57(0.86-2.85) | 0.136 | 1.40(0.70-2.81) | 0.335 |
| **Ridge width(mm)** | 2.92(1.87-4.55) | **<0.0001***** | 2.92(1.86-4.57) | **<0.0001***** |
| **Glycaemic Control (HbA1c<7 Vs (HbA1c≥7)** | 0.90(0.50-1.64) | 0.752 | 0.52(0.24-1.11) | 0.089 |

**Note:** CI = confidence interval; HbA1c = glycated hemoglobin. Dependent variable was implant-diameter allocation selection (4.1 mm vs. 3.3 mm reference). Univariate and multivariate estimates were calculated using logistic regression analyses. a Reference category is Male. b Reference category is HbA1c ≥7%..* p < 0.05, ** p < 0.01, *** p < 0.001.
