# Supplementary material for: Stratified Randomized Controlled Trial of ∅3.3 mm Versus ∅4.1 mm Hydrophilic Titanium‐Zirconium Implants Supporting Posterior Mandibular Fixed Prostheses in Hyperglycaemic Subjects
Source: Clin Oral Implants Res. 2026 Sep 24;37(11):1328–49. doi: 10.1111/clr.70175 (PMC13743795; doi:10.1111/clr.70175)
Supplement: Supplementary file 2 [file CLR-37-1328-s002.docx]

| **Parameter** | **HbA1c ≥7**  **n= 41 patients/ 82 implants** | **HbA1c <7**  **n=42 patients,**  **84 implants** | **P value** |
| --- | --- | --- | --- |
| **Marginal Bone Loss- Median (IQR)** | | | |
| MBL_mesial_T3 | 0.25(0.05-0.63) | 0.08(-0.09-0.31) | **0.0016^M^*** |
| MBL_distal_T3 | 0.36(0-0.7) | 0.03(-0.25-0.19) | **<0.0001^M^**** |
| MBL_average_T3 | 0.29(0.08-0.55) | 0.04(-0.14-0.26) | **<0.0001^M^**** |
| MBL_mesial_T4 | 0.42(0.2-0.89) | 0.09(-0-0.46) | **<0.0001^M^**** |
| MBL_distal_T4 | 0.6(0.25-0.88) | 0.07(-0.06-0.36) | **<0.0001^M^**** |
| MBL_average_T4 | 0.53(0.19-0.87) | 0.07(-0.06-0.36) | **<0.0001^M^**** |
| **Frequency of implants presenting with MBL average ≥0.5mm** | | | |
| **At 6 months**  <0.5mm  >=0.5mm | 56(68.3)  26(31.7) | 79(94.05)  5(5.95) | **<0.0001^c**^** |
| **At 12 months**  <0.5mm  >=0.5mm | 40(48.7)  42(51.2) | 73(86.9)  11(13.1) | **<0.0001^c**^** |
| **Implant Stability- Median (IQR)** | | | |
| ISQ_Avg_T0 | 73.1(68.1-79) | 70(66.4-75.8) | **0.025*** |
| ISQ_Avg_T2 | 76.4(73.6-80.5) | 79(76-80.6) | **0.034*** |
| Difference | 3.5(0-7) | 8.16(3.58-11.6) | **0.0001**** |
| **Clinical Peri-implant indices-Median (IQR)** | | | |
| Average PI_T3 | 0.25(0-0.5) | 0.25(0-0.5) | 0.7641^M^ |
| Average_GI_T3 | 0(0-0.25) | 0(0-0.25) | 0.0601^M^ |
| Average PD_T3 | 2.5(2.25-2.75) | 2.5(2.13-2.75) | 0.3718^M^ |
| Average PI_T4 | 0.25(0-0.75) | 0.25(0-0.5) | 0.2788^M^ |
| Average_GI_T4 | 0.25(0-0.5) | 0(0-0.25) | **0.0116^M^*** |
| Average PD_T4 | 2.5(2-3) | 2.25(2-2.5) | 0.1934^M^ |
| **Healing Index** | | | |
| HI at T1 | 4(4-5) | 5(4-5) | 0.002* |
| HI at T2 | 5(4-5) | 5(5-5) | 0.4819 |

Supplement table 4: Subgroup analysis comparing participants with **HbA1c ≥7% and HbA1c<7%** for primary and secondary outcome measures.

M-Mann Whitney U test., c-Chi square test
