# Supplementary material for: Stratified Randomized Controlled Trial of ∅3.3 mm Versus ∅4.1 mm Hydrophilic Titanium‐Zirconium Implants Supporting Posterior Mandibular Fixed Prostheses in Hyperglycaemic Subjects
Source: Clin Oral Implants Res. 2026 Sep 24;37(11):1328–49. doi: 10.1111/clr.70175 (PMC13743795; doi:10.1111/clr.70175)
Supplement: Supplementary file 3 [file CLR-37-1328-s003.docx]

**Supplement Table 1. PICOT of the study**

| Patients | Patients with Varying levels of hyperglycaemia (stratified into HbA1c < 7.0, and HbA1c ≥ 7.0) with mandibular posterior edentulous situation who require fixed dental prosthesis. |
| --- | --- |
| Intervention | Two ⌀3.3mm hydrophilic Ti-Zr implants supporting a two/three-unit FDP |
| Control | Two ⌀4.1mm hydrophilic Ti-Zr implants supporting a two/three-unit FDP |
| Outcome | Success rates (Buser et al., 1990),  Marginal bone level changes (MBL) (primary end point)  Peri-implant indices, Implant Stability Quotient (ISQ)  Healing Index of Landry et al (Landry RG, 1988; Masse et al., 1993)  Biologic complications such as infection, wound breakdown  Technical complications such as prosthetic fracture  Patient Reported Outcome Measures (PROMs) by OHIP-14 (Slade, 1997) |
| Time | T0 = At implant placement (Implant Stability Quotient- ISQ, Initial Depth of Implant Placement- IDIP)  T1 = At 2 weeks following implant placement (Healing Index)  T2 = At 3 (±1) months following implant placement (ISQ, Healing Index)  T3 = At 6 (±1) months following prosthesis delivery (MBL, Peri-implant indices)  T4 = At 12 (±1) months following prosthesis delivery (MBL, Peri-implant indices, PROMs) |

Buser, D., Weber, H. P., & Lang, N. P. (1990). Tissue integration of non-submerged implants. 1-year results of a prospective study with 100 ITI hollow-cylinder and hollow-screw implants. *Clin Oral Implants Res*, *1*(1), 33-40. <https://www.ncbi.nlm.nih.gov/pubmed/2099210>

Landry RG, T. R., Howley T. (1988). Effectiveness of benzydamyne HCl in the treatment of periodontal post-surgical patients. *Research in Clinic Forums.*, *10*, 105-118.

Masse, J. F., Landry, R. G., Rochette, C., Dufour, L., Morency, R., & D'Aoust, P. (1993). Effectiveness of soft laser treatment in periodontal surgery. *Int Dent J*, *43*(2), 121-127. <https://www.ncbi.nlm.nih.gov/pubmed/8320005>

Slade, G. D. (1997). Derivation and validation of a short-form oral health impact profile. *Community Dent Oral Epidemiol*, *25*(4), 284-290. <https://doi.org/10.1111/j.1600-0528.1997.tb00941.x>
