# Supplementary material for: Stratified Randomized Controlled Trial of ∅3.3 mm Versus ∅4.1 mm Hydrophilic Titanium‐Zirconium Implants Supporting Posterior Mandibular Fixed Prostheses in Hyperglycaemic Subjects
Source: Clin Oral Implants Res. 2026 Sep 24;37(11):1328–49. doi: 10.1111/clr.70175 (PMC13743795; doi:10.1111/clr.70175)
Supplement: Supplementary file 4 [file CLR-37-1328-s004.docx]

**Supplement Figure 1:** Box plot displaying the ISQ changes in ⌀3.3mm and ⌀4.1mm implants at the baseline (T0) and 3-month post implant placement (T2) time points in the HbA1c<7% and HbA1c≥7% sub-groups.
