# Supplementary material for: Stratified Randomized Controlled Trial of ∅3.3 mm Versus ∅4.1 mm Hydrophilic Titanium‐Zirconium Implants Supporting Posterior Mandibular Fixed Prostheses in Hyperglycaemic Subjects
Source: Clin Oral Implants Res. 2026 Sep 24;37(11):1328–49. doi: 10.1111/clr.70175 (PMC13743795; doi:10.1111/clr.70175)
Supplement: Supplementary file 5 [file CLR-37-1328-s005.docx]

**Supplement Table 3: Multivariable logistic regression analysis assessing covariates associated with Glycaemic Control (HbA1c<7 vs HbA1c≥7.0%).**

| **Parameter** | **Univariate** |  | **Multivariate** |  |
| --- | --- | --- | --- | --- |
|  | **Odds(95%CI)** | **P value** | **odds(95%CI)** | **P value** |
| **Age(per year Increase)** | 1.09 (1.05-1.12) | **<0.0001***** | 1.09 (1.06-1.13) | **<0.0001***** |
| **Sex(Female Vs Male)** | 2.51 (1.37-4.62) | **0.003**** | 2.74 (1.36-5.53) | **0.005**** |
| **Ridge width(mm)** | 1.30 (0.98-1.72) | 0.060 | 1.50 (1.05-2.14) | **0.025*** |
| **Implant Diameter(4.1vs3.3)** | 0.90 (0.50-1.064) | 0.752 | 0.45 (0.20-0.98) | 0.045 |

**Note**: CI = confidence interval; HbA1c = glycated hemoglobin. Dependent variable is glycemic control status (HbA1c <7 vs. HbA1c ≥7 reference). Univariate and multivariate estimates were calculated using logistic regression analyses. a Reference category is Male. b Reference category is 3.3 mm Implant Diameter. * p < 0.05, ** p < 0.01, *** p < 0.001.
